# Aberrant Synaptic Pruning in CNS Diseases: A Critical Player in HIV-Associated Neurological Dysfunction?

**DOI:** 10.3390/cells11121943

**Published:** 2022-06-16

**Authors:** Zachary Watson, Shao-Jun Tang

**Affiliations:** 1 Department of Neuroscience and Cell Biology, University of Texas Medical Branch, Galveston, TX 77555, USA; zawatson@utmb.edu; 2 Stony Brook University Pain and Analgesia Research Center (SPARC), Department of Anesthesiology, Renaissance School of Medicine, Stony Brook University, Stony Brook, NY 11794, USA

**Keywords:** HIV, glia, synapses, pain, microglia, astrocytes

## Abstract

Even in the era of effective antiretroviral therapies, people living with Human Immunodeficiency Virus (HIV) are burdened with debilitating neurological dysfunction, such as HIV-associated neurocognitive disorders (HAND) and HIV-associated pain, for which there are no FDA approved treatments. Disruption to the neural circuits of cognition and pain in the form of synaptic degeneration is implicated in developing these dysfunctions. Glia-mediated synaptic pruning is a mechanism of structural plasticity in the healthy central nervous system (CNS), but recently, it has been discovered that dysregulated glia-mediated synaptic pruning is the cause of synaptic degeneration, leading to maladaptive plasticity and cognitive deficits in multiple diseases of the CNS. Considering the essential contribution of activated glial cells during the development of HAND and HIV-associated pain, it is possible that glia-mediated synaptic pruning is the causative mechanism of synaptic degeneration induced by HIV. This review will analyze the known examples of synaptic pruning during disease in order to better understand how this mechanism could contribute to the progression of HAND and HIV-associated pain.

## 1. Introduction

As HIV has transitioned to a chronic disease with the implementation of effective antiretroviral therapies, people living with HIV (PLWH) are living longer, healthier lives. However, PLWH can still be affected by the often-debilitating neurological dysfunction associated with HIV. HIV-associated neurocognitive disorders (HAND) and HIV-associated chronic pain affect ~42.6% [1] and 55–67% [2] of PLWH, respectively, even in the era of combined antiretroviral treatment (cART). HAND is characterized by cognitive slowing, memory and attention deficits, and is diagnosed via deficits in cognitive domains determined by neuropsychological testing. The Frascati criteria divide HAND into three subdivisions based on the number of deficits and level of functional impairment, escalating in severity as follows: asymptomatic neurocognitive impairment (ANI), mild neurocognitive impairment (MNI), and HIV-associated dementia (HAD) [3]. The most severe subtype, HAD, decreased in prevalence with the introduction of HAART; however, the overall prevalence of HAND remains the same. HIV-associated pain is a chronic pain state characterized by paresthesia, dysesthesia, hyperalgesia, and allodynia occurring in a “stocking and gloves” pattern [4]. HIV infection affects the entire nervous system, but dysfunction in specific nervous system regions is associated with HAND and HIV-associated pain. Dysfunction in multiple brain regions has been associated with HAND [5], and historically, the hippocampus and cortex have been studied due to their importance in memory and cognition, respectively [6]. The pathology of HIV-associated pain has been seen in the peripheral and central nervous system. Peripheral nerve and spinal cord pathology have been confirmed to contribute to HIV-associated pain [7,8], but there are also areas of the brain involved in pain perception, such as the CA1 hippocampus [9] and nucleus accumbens [10], which could also contribute to this pain phenotype.

HAND and HIV-associated pain are characterized by multiple different pathologies in the CNS, some of which are common to both forms of dysfunction. Neuronal loss and HIVE are both associated with HAD and higher viral load, but not with the less severe forms of HAND, which have become more prevalent in the cART era [11,12]. BBB disruption is known to occur during HIV infection and has been linked to HAND in a microarray experiment but requires further investigation to establish a causal link [12]. Sensory neuropathy, peripheral inflammation, and epidermal nerve dieback are peripheral pathologies of HIV-associated pain [7]. Neuroinflammation and neurodegeneration are the pathologies of HIV-associated pain in the CNS [4,11], which are also shared with HAND. Synaptic degeneration, a form of neurodegeneration, has been found to correlate with every level of HAND impairment [13] and is implicated in the development of HIV-associated pain [14]. This review will consider the contribution of synaptic degeneration to HIV-associated neurological dysfunction, examine glia-mediated synaptic pruning in the context of disease of the CNS, and theorize a possible role for pruning in HIV-driven synapse loss and dysfunction, including hypothetical mechanisms.

## 2. Synaptic Degeneration Associated with HAND and HIV-Associated Pain

### 2.1. Synaptic Degeneration and HAND

Several of the pathologies associated with HAND were present in the most severe form, HAD, and not ANI and MNI, which now comprise 96% of the HAND impairment seen in HIV patients. A comparison of cortical synaptic density in HIV patients with a range of neuropsychological impairments found a correlation between decreasing excitatory synaptic density and increasing impairment at all levels, not just in HAD [15]. Synaptic degeneration induced by HIV is not just limited to excitatory synapses. A study of GABAergic mRNAs representative of inhibitory synapses (GAD1, GAD2, GJD2) in HIV brain tissue found that all GABAergic mRNAs analyzed were decreased in both cART treated low-HIV-replication patients and non-cART-treated high-HIV-replication patients, as compared to HIV seronegative patients [16]. This corresponds to the view that HIV-induced neurological damage and dysfunction can be independent of viral replication, which is important in the post-cART era, where viral replication is not present or at very low levels in patients. Non-invasive imaging studies in PLWH utilizing fMRI have found decreased connectivity between brain regions corresponding with HAND, which could be indicative of synaptic degeneration [17]. Synaptic degeneration is known to contribute to the neurocognitive decline in diseases, such as Alzheimer’s Disease (AD) [18], Parkinson’s Disease [19], Huntington’s Disease [20], and West Nile Virus infection (WNV) [21]. It is likely a key mechanism in the development of HAND.

### 2.2. Synaptic Degeneration and HIV-Associated Pain

A study investigating the lumbar spinal dorsal horn tissue of HIV patients found that pain expression was associated with a decrease in synaptic markers, indicating synaptic degeneration. Moreover, the pain-positive patients also increased HIV glycoprotein gp120, glial activation, and inflammation [14]. All synaptic markers tested (PDS-95, NR1, Synapsin 1) were significantly decreased in pain-positive HIV patients compared to pain-negative patients and HIV-negative patients. Similar to how synaptic degeneration can lead to cognitive dysfunction in the brain, it is likely that disruption to the pain processing circuitry of the spinal dorsal horn (SDH) would result in abnormal pain syndromes. The pain processing circuitry of the spinal dorsal horn comprises primary and secondary afferents and secondary projection neurons and inter-neurons that modulate the activity of these pain-transmission neurons [22]. Inhibition of excitatory interneurons and excitation of inhibitory interneurons are critical to maintaining the strong inhibition that counteracts the development of hyperexcitability and central sensitization. Therefore, synaptic degeneration in the spinal dorsal horn could represent a crucial mechanism in the development of central sensitization and chronic pain through the loss of both excitatory and inhibitory connections [23]. Central sensitization is a pain state where afferent neurons of the spinal cord are in a state of hyperexcitability, leading to the amplification of noxious signals (hyperalgesia) and recruitment of normally sub-threshold afferents, leading to non-noxious stimuli resulting in pain perception (allodynia) [24]. Another consequence of disinhibition in the SDH is the un-silencing of interneurons that polysynaptically connect non-nociceptive primary afferents to secondary nociceptive afferents leading to allodynia [25]. The importance of synaptic degeneration in the development of chronic pain in HIV patients is clear; however, specific mechanisms of how synapses are lost remain to be investigated.

### 2.3. What Is Causing Synaptic Degeneration in HIV Patients?

While HAND and HIV-associated pain are two separate sequelae of HIV infection of the CNS, synapse degeneration has been implicated in the development of both syndromes, suggesting that they could share this causative mechanism. Loss of synapses in the CNS can be attributed to neuronal loss, disruption to synapse maintenance, neuron-autonomous homeostatic scaling, and non-autonomous glia-mediated pruning. As previously mentioned, synapse degeneration precludes neuronal loss during the development of HAND [15], and as such, neuronal loss is not likely to be causative of the synaptic degeneration seen in all but the latest stage of HAND. Neurons are some of the longest-lived cells in the body, and the maintenance of individual organelles and structures is important for their continued survival. Synapses (in the CNS) are also long-lived and are critical to neuronal networks, so when synapse maintenance mechanisms, such as endomembrane degradation and Ca^2+^ homeostasis, are disrupted, this can lead to synapse loss and neurodegeneration [26,27]. Neuron-autonomous homeostatic scaling refers to synapse remodeling as a part of structural plasticity, in which apoptosis and necrosis pathways are triggered locally in synapses as a form of long-term depression and associated with structural retraction [28]. Scaling could occur during HIV neurological dysfunction to protect neurons after synaptic damage from the direct and indirect effects of neurotoxic proteins, leading to synapse degeneration [29]. Glia-mediated pruning refers to the phagocytosis and elimination of synapses by astrocytes and microglia. Reactive microglia and astrocyte cells are an important feature of HIV infection [30,31], and further, astrogliosis was linked to pain in HIV patients [14]. While the other mechanisms of synapse loss could play a role in HAND and HIV-associated pain, the importance of glia in these disorders necessitates the further study of glial synaptic pruning during disease, which will be the focus of this review. This mechanism could directly contribute to HIV-induced synapse degeneration, and investigating this causative mechanism could lead to breakthroughs in the treatment of these disorders.

## 3. Glia-Mediated Synaptic Pruning in the Normal CNS

### 3.1. Discovery and Overview of Glia-Mediated Synaptic Pruning

The removal of synaptic connections in a controlled manner is an important aspect of structural plasticity in the healthy CNS [32,33] and plays an important role in the reorganization of neuronal networks. Both microglia and astrocytes contribute to synaptic pruning; however, microglial synapse pruning was first reported in the developing hippocampus of mice [34]. Synaptic pruning by astrocytes was first seen in the retinogeniculate system in mice, a model system for activity-regulated neuronal circuit plasticity. During the development of the dorsal lateral geniculate nucleus (dLGN), excessive synaptic inputs are initially formed but are removed later. It was found that both microglia [35] and astrocytes [36] engulf and prune the supernumerary synapses of the retinal ganglion cells (RGC), projecting into the dLGN. Glial synaptic engulfment is commonly quantified via immunofluorescent staining of a sample for a synaptic marker and a glial cell marker, which is then imaged via a confocal microscope and analyzed with 3D image reconstruction software for the presence of synaptic material inside of glial cells. [37]. This form of colocalization analysis does not go as far as confirming that phagocytosis has taken place, which requires the additional staining of a lysosomal marker alongside the synaptic marker and glial marker. So-called “triple-positive-puncta” that mark the colocalization of all three markers indicate that synaptic material has entered into the endolysosomal degradation pathway of a glial cell.

Synaptic pruning by glial cells is not limited to the dLGN or only limited to the developmental period. It has also been seen in multiple locations that we currently know of in the developing CNS, such as the hippocampus, cortex, and thalamus [38]. Beyond the developmental period, Chung, et al. saw that pruning by astrocytes in the adult somatosensory cortex and engulfment of synapses by microglia in the adult CA1 hippocampus was crucial in contextual fear memory formation and forgetting in mice [39]. Pruning activity has also been seen in the spinal cord; in one study, increased complement levels accompanied synapse loss [40], and in another, a synaptic marker was found inside of spinal cord microglia [41]. In addition to physiological states, disruption to the homeostatic level of synapse pruning is postulated to contribute to disorders, such as schizophrenia and autism [42].

### 3.2. Microglial Mechanisms of Synaptic Pruning

**Figure 1:**
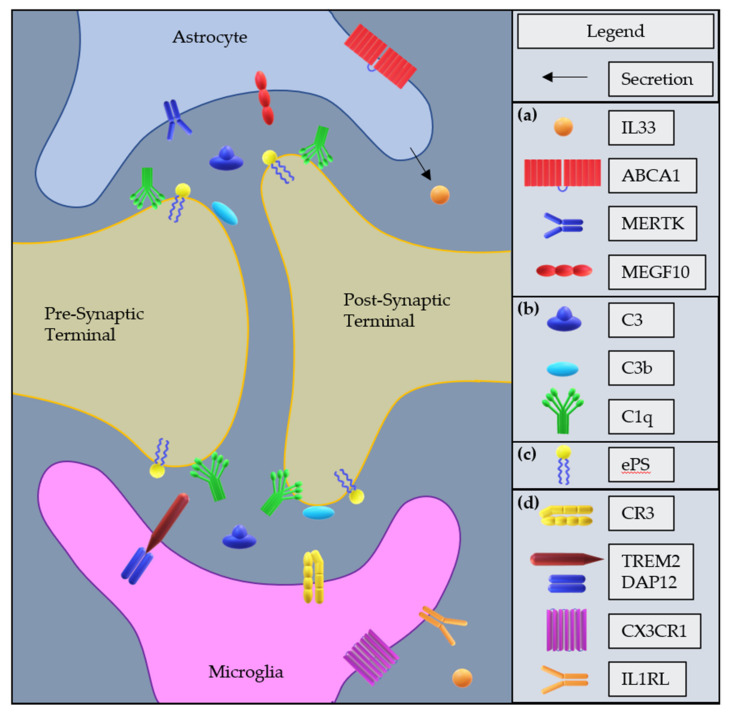
Potential molecular mechanisms of microglia- or astrocyte-mediated synaptic pruning: (**a**) Astrocyte factors mediating synaptic pruning activity; IL33 is secreted by astrocytes and upregulates the pruning activity of microglia, ABCA1 is an upstream modulator of MEGF10, MERTK and MEGF10 are phagocytosis receptors that recognize “eat-me” signals. (**b**) Complement components mediating synaptic pruning, including intermediate component C3 which is cleaved into C3b and C3a; C3b and C1q are opsonins that mark synapses for phagocytosis. (**c**) Externalized Phosphatidylserine “eat-me signal”. (**d**) Microglial receptors mediating synaptic pruning activity; CR3 is a phagocytosis receptor that recognizes C3b fragment iC3b; TREM2 bound to DAP12 is a receptor of a wide range of ligands that modulates microglial activation, proliferation, and phagocytosis; CX3CR1 is a chemokine receptor which promotes microglial recruitment and modulates the pruning activity of microglia; IL1RL is a receptor for IL33 which upregulates the pruning activity of microglia.

There are multiple known molecular mechanisms of microglia-mediated synapse pruning that have been identified [See Figure 1]. These include several complement system components (C1q, C3, and CR3). The complement system comprises several cascading signaling pathways that facilitate the destruction and removal of pathogens and other threats by macrophages and antibodies [43]. It was discovered that the complement system, and specifically C1q and C3, were necessary for developmental synapse elimination in the mouse dLGN [44]. C1q is the initiator of the classical complement cascade and C3 is a critical molecule in both the classical and alternative complement cascade. Both C1q and C3 fragment C3b function as “eat me signals” and will opsonize developing synapses marking them for phagocytosis [44]. In a further study, it was confirmed that global knockdown of C3, global knockdown of CR3, and pharmacological attenuation of microglial activity with minocycline decreased the pruning activity of microglia and impaired synapse remodeling in the dLGN [35]. Complement receptor 3 (CR3) is only expressed on microglia and infiltrating macrophages in the CNS and recognizes the C3b fragment iC3b triggering phagocytosis of opsonized material. CX3CR1 is a chemokine receptor expressed only on microglia in the CNS and the binding of its only known ligand, fractalkine (CX3CL1), promotes microglial recruitment during the development of the hippocampus [34], and global knockdown of CX3CR1 and CX3CL1 showed that they are required for microglial pruning of synapses in the developing barrel cortex [45,46]. The downstream molecular mechanisms induced by CX3CR1 in microglia, leading to synaptic pruning, remain to be elucidated. Externalized phosphatidylserine (ePS) is involved in caspase-3-mediated apoptosis, and likely functions in conjunction with C1q to label synaptic inputs for engulfment and elimination by microglia in the developing mouse dLGN [47]. Triggering Receptor Expressed on Myeloid Cells 2 (TREM2) is a membrane-bound protein with an extracellular Ig-like type V domain, which functions as a receptor for a wide range of ligands and is expressed only on microglia in the CNS [48]. TREM2 global knockout decreased synaptic pruning and increased dendritic spine density in the hippocampus of developing mice [49]. This effect is likely due to the important roles of TREM2 in both induction of phagocytosis through ligand binding and cell activation [48,49,50]. To investigate mechanisms leading to pain, it is important to note that the TREM2 receptor has also been implicated in the development of pain through central mechanisms in the spinal cord. A TREM2-activating antibody induced pain in a mouse model, and when TREM2 expression was globally knocked down in a cisplatin-induced pain model, pain was attenuated [51,52]. This suggests a possible role for TREM2-mediated synaptic pruning in the spinal cord leading to pain. 

### 3.3. Astrocyte Mechanisms of Synaptic Pruning

In astrocytes, confirmed mechanisms [See Figure 1] include MEGF10 [36], Mertk [36], and ABCA1 [53]. Both MEGF10 and Mertk are cell surface phagocytosis receptors that recognize “eat me” signals, such as C1q [54]. MEGF10 is localized on astrocyte cell membranes, while Mertk is present on multiple cell types. In the developing mouse dLGN, astrocyte engulfment of synaptic inputs from RGCs was observed, and the involvement of MEGF10 and Mertk pathways was confirmed through global knockdown of these genes, separately and together. In each knockdown condition, the synaptic pruning activity of astrocytes was significantly decreased, and there were excess synapses present in the dLGN [36]. Interestingly, when the relative abundance of microglia and astrocytes was taken into account, the synaptic pruning capacity of astrocytes in the dLGN was much higher than microglia [36]. ABCA1 is known to be required for astrocyte phagocytosis of extracellular debris after brain injury and likely does so as an upstream modulator of MEGF10 activity [53,55].

### 3.4. Interactions between Microglia and Astrocytes Mediating Synaptic Pruning

Recent studies have demonstrated that astrocytes and microglia communicate with and influence the activity of each other [56], and this finding has been extended to the synaptic pruning activity of these cells. A study of developmental pruning in a TREM2 knockout mouse model discovered increased astrocyte phagocytosis in the synapses. This study utilized mice with a global knockdown of TREM2 expression and found decreased synapse levels across multiple brain regions at one month of age. At the same time, they examined the synaptic pruning activity of glia and found that microglial pruning was decreased while astrocyte pruning was increased [57]. This shows that in normal developmental conditions, microglia exhibit a form of control, limiting the synaptic pruning activity of astrocytes, and suggests that the pruning activities of these two glial cells are interconnected. Astrocytes have been seen to influence microglial synaptic pruning via the expression and secretion of IL-33 [Figure 1]. Investigators found that conditional knockdown of IL-33 in astrocytes resulted in increased synapse levels and that injection of IL-33 into the spinal cord increased microglial pruning, leading to synapse depletion, which was reversed by conditional knockdown of IL1RL (a receptor for IL-33) in microglia [41]. This finding demonstrates that, in addition to microglia affecting the synaptic pruning activity of astrocytes, astrocytes instruct the pruning activity of microglia during development. Knowing that inter-glial communication can affect synaptic pruning during development, it is important to consider the effect and potential contribution of both cell types to synaptic pruning during disease.

## 4. Glia-Mediated Synaptic Pruning in CNS Diseases

### 4.1. The Role of Glia in Alzheimer’s Disease

Alzheimer’s disease is one of the most common neurodegenerative diseases, leading to memory loss and cognitive decline. AD is characterized by two types of misfolded protein aggregates: hyperphosphorylated tau neurofibrillary tangles and amyloid-beta plaques. Synaptic degeneration is a prominent pathology in AD [58] and is the anatomical pathology that best correlates with cognitive decline in AD patients [18,59]. Microglia and astrocyte cells have been suggested to play a two-sided role in AD. Activated microglia and reactive astrocytes contribute to an inflammatory environment but also enclose and phagocytose plaques in AD [60,61,62]. This suggests that glia could protect against the deleterious effects of late-stage protein aggregates but contribute to persistent inflammation. 

#### 4.1.1. Microglia-Mediated Synaptic Pruning in AD

The importance of microglia is highlighted by the role of TREM2, rare variants of which, most notably the R47H mutation, increase the risk of developing AD by 2–4-fold [63]. As previously mentioned, TREM2 is required for microglial synapse pruning as well as proliferation and activation. The contribution of TREM2 to AD progression is complex, but one recent study shows decreased microglial synapse engulfment in a pure-tauopathy PS19 mouse model and patient AD brains, both expressing the R47H variant of TREM2 [64]. This suggests that malfunction of microglia-mediated synaptic pruning may contribute to AD development. However, this proposal needs to be validated by considering the contribution of decreased inflammation seen in this model. Classical complement C1q-mediated microglial synapse degeneration was also seen in a pre-plaque amyloid mouse model (human amyloid precursor protein-expressing J10 mouse), and pruning of synapses by microglia was confirmed to occur in WT mice in response to oligomeric amyloid-beta [65]. This microglial synaptic pruning was confirmed to be mediated by C1q and C3 via global knockdown and by CR3 via conditional knockdown in microglia [65]. Oligomers of tau and amyloid-beta are precursor protein aggregates to the much larger fibrils and plaques but are the most toxic form of these proteins. There are multiple mechanisms by which oligomeric amyloid-beta exhibits synaptotoxicity [66], and so this toxic protein may be directly inducing microglia to increase their synaptic pruning activity, though it is more likely that an increase in the number of damaged synapses leads to increased microglial pruning. 

#### 4.1.2. Astrocyte-Mediated Synaptic Pruning in AD

Another gene linked to increased risk of AD development is APOE, with APOE2 decreasing risk 2-fold [67] and APOE4 increasing risk 12-fold [68]. A study of APOE variants found that APOE2 knock-in increased the synaptic pruning capability of astrocytes and APOE4 decreased synaptic pruning capability [69]. This finding reinforces the hypothesis that synaptic pruning by both astrocytes and microglia is a protective mechanism in AD. Phagocytosis of synapses from diseased neurons could limit the spread of toxic self-seeding tau and amyloid oligomers [70,71]. However, this protective function may become deleterious as synapse loss accumulates. Contributions of microglia to astrocyte synaptic pruning in AD and vice versa have not been investigated but merit further research, especially given the importance of TREM2 in AD and the function of TREM2 in mediating microglia–astrocyte interaction in developmental pruning.

### 4.2. The Role of Glia in West Nile Virus CNS Infection

West Nile Virus (WNV) is a neurotropic virus that causes encephalitis and meningitis in the CNS and long-lasting cognitive deficits. More than half of those infected with WNV experienced cognitive deficits up to a year after infection [21]. WNV can infect neurons, microglia, and astrocytes but induces apoptosis preferentially in neurons [72]. Astrocytes and microglia are activated during WNV infection, with microglia assuming a predominantly pro-inflammatory phenotype. Proinflammatory cytokines, such as IL-1β, -6, -8, and TNF-α, are upregulated during WNV infection [73]. However, in a pharmacological microglia depletion mouse model of WNV infection, RNA levels of other inflammatory cytokines and chemokines (CCL2, CCL3, CCL5, CCL7, CXCL9, CXCL10, and IFN-γ) did not decrease compared to control WNV infected mice, indicating that microglia alone are not responsible for the inflammatory environment present in WNV [74]. In addition, brain viral titer increased, and mortality dramatically increased in microglia-depleted mice [74]. This suggests that damage induced by proinflammatory microglia is secondary to the overall protective effect of microglia in the CNS during WNV infection. Synaptic degeneration was seen in WNV encephalitis patient CA3 and CA1 hippocampal regions, a recapitulated pathology in the CA3 hippocampus of mice intracranially infected with an attenuated WNV strain [75].

#### Microglia-Mediated Synaptic Pruning in WNV

Synaptic engulfment analysis of glial cells in this model revealed that microglia, and not astrocytes, engulfed synapses in the CA3 hippocampus. Knockdown of C3 and C3aR rescued synapse levels and decreased microglial pruning activity, indicating that the classical complement pathway is integral to this process [75]. C3aR is expressed by microglia and neurons in the CNS, and it was postulated that C3aR on microglia recognizes C3 cleavage products localized to synapses, leading to phagocytosis in that model. A separate study found that following recovery from WNV, the presynaptic loss that correlated to memory deficits persisted in WNV-infected mice, and microglial engulfment of presynaptic puncta was linked to CD8 t-cell expression of IFN-γ [76]. Interestingly, post-synaptic markers were not decreased in patient or mouse tissue. The specific loss of pre-and not post-synaptic termini suggests that either WNV causes damage to pre-synaptic termini at a much greater rate than to post-synaptic termini, causing the preferential engulfment, or that WNV induces microglia to target pre-synaptic termini specifically. The overall protective role of microglia during WNV infection implies that the elimination of synapses could serve a protective purpose, such as possibly limiting the transsynaptic spread of viral particles [77], with the side effect of accumulated synaptic degeneration and cognitive deficits.

### 4.3. The Role of Glia in Zika Virus CNS Infection

Zika virus (ZIKV) infection causes neurological issues in adults as well as severe neurodevelopmental deficits, including, most notably, microcephaly in infants. Similar to WNV infection, ZIKV infects all three cell types but appears to target neurons over microglia and astrocytes [78] preferentially. During ZIKV infection, there is increased microglial activation. Although astrocyte activation has not been reported, astrocytes contribute to neuroinflammation during ZIKV infection and are suggested to play a protective role through IFN-1 signaling, limiting viral spread [79]. 

#### 4.3.1. Microglia-Mediated Synaptic Pruning in ZIKV

Microglial synapse pruning has been observed during ZIKV infection. In an intracranial ventricular injection mouse model of ZIKV infection, presynaptic degeneration and synaptic dysfunction were detected in the CA3 hippocampus alongside memory deficits assessed using a novel object recognition (NOR) test [78]. Meanwhile, microglial engulfment of presynaptic terminals was also increased compared to control in the CA3 region. Other hippocampal regions were unaffected, and total hippocampal homogenate did not have differences in synaptic marker levels. Neither ZIKV viral load nor neuronal damage (assessed using FlouroJade B) was increased in the CA3 compared to regions without synapse loss [78], suggesting that synaptic degeneration was the result of functional differences in microglial activity in the CA3.

The blocking of microglial activation using minocycline prevented ZIKV-induced memory deficits and did not lead to increased brain viral replication [78]. This suggests that activated microglia are not essential for mitigating the spread of ZIKV in the brain. Presynaptic degeneration and microglial engulfment were reversed in ZIKV-infected mice by blocking TNF-α signaling or by neutralizing c3 or C1q-soluble protein in the brain with matching antibodies [78]. Microglial engulfment of post-synaptic and neuronal cell body material was seen in ZIKV in the same post-recovery study referenced above in WNV, which linked ZIKV-induced microglial post-synaptic pruning and neuronal apoptosis to IFN-γ [76]. This study also used an intracranial injection model of ZIKV neuroinvasion but examined later timepoints for synaptic- and neuro-degeneration. While one study found only pre-synaptic degeneration and the other found only post-synaptic degeneration induced by ZIKV in the CA3 region, this contradiction could be due to the different time points utilized with the first study investigating early infection up to day-post-infection (d.p.i.) 6 and late recovery at 60 d.p.i., while the second study examined synapses at d.p.i. 7, 25, and 52. This time-based differential targeting of synaptic termini could also be induced by IFN-γ, as observed in the second study; however, there were differences in the ZIKV strain used and other differences that bar a direct comparison between the two studies [76,78].

#### 4.3.2. Astrocyte-Mediated Synaptic Pruning in ZKV

An increase in astrocyte phagocytosis of ZIKV viral particles marked with nanogold was demonstrated via ultra-structural analysis after pharmacological microglia depletion in a mouse model, in which mice deficient in type 1 interferon signaling were injected intravenously with ZIKV [80]. This finding suggests that astrocytes might have a compensatory role in disease-associated phagocytosis and possibly pruning in response to ZIKV infection.

### 4.4. Overview of Glia-Mediated Synaptic Pruning during Disease

The most common mechanism reported in disease-induced microglial and astrocytic synaptic pruning is the complement pathway [Table 1], which has also been identified as a crucial element of healthy synaptic pruning. Complement components and receptors are responsible for the opsonization and recognition of synapses during pruning. This conservation of functional mechanisms leads us to postulate that the difference between disease-induced “aberrant” synaptic pruning and physiological synaptic pruning lies in the induction of the complement pathway and activation of microglia and astrocytes. If, as hypothesized in the case of AD and WNV, microglial synaptic pruning during disease is an initially protective function, then it would be expected that only damaged or diseased synapses would be targeted for elimination. Discovering how increased expression of the complement pathway is induced and what criteria result in a synapse being marked for pruning will be necessary, in order to understand the functional difference between physiological and diseased-state synaptic pruning. 

## 5. Does Dysregulated Synaptic Pruning Contribute to HIV-Associated Neurological Disorders?

### 5.1. The Role of Glia in HIV CNS Infection and Their Potential Role in Synaptic Pruning

Glial cells are the primary cells infected by HIV in the CNS, while neurons are not [31,84]. The neurotoxic effects of HIV are, therefore, mediated indirectly by infected glia and their secreted viral proteins. In the era of combination antiretroviral therapy (cART), HIV infection has become a chronic condition. Although HIV replication can be controlled by cART at a barely detectable level, individual viral genes can still be transcribed and translated because drugs do not inhibit transcription and translation in cART. Hence, HIV proteins can still be expressed and secreted in the presence of cART drugs [14,85]. Individual neurotoxic viral proteins, such as Tat and gp120, have been suggested as the major etiological causes for HAND [86] and HIV-associated pain [14] and used for generating relevant animal models that have shed light on the underlying pathogenic mechanisms. Tat and gp120 exhibit “direct” neurotoxicity through the induction of cellular damage pathways after binding to receptors on the surface of neurons. The binding and internalization of Tat is mediated by the low-density lipoprotein-receptor-related protein (LRP) [87], and gp120 binds to chemokine coreceptors, such as ccr4 and cxcr5 [88]. Both proteins cause excitotoxicity and calcium imbalance through the activation of the NMDA receptor [89,90,91], as well as inducing downstream damage pathways [13,29]. Both microglia and astrocytes are activated in gp120- [92] or Tat-based animal models [93], likely due to both neuronal damage and the effect of these proteins on microglia and astrocyte cells [94]. Because, as shown in the diseases discussed above, these types of glia prune synapses during the pathogenesis, it is tempting to hypothesize that microglia and astrocytes might also contribute to the HIV-associated dysfunction of neuronal circuits via dysregulated synapse pruning. This working hypothesis is consistent with the results from recent studies that show an essential role of microglia in synaptic degeneration in the spinal dorsal horn of gp120 mouse models of HIV-associated pain [94] and in the engulfment of synapses coinciding with synaptic degeneration in a Tat model of HAND, which commonly expresses learning deficits [93,95,96]. Ru et al., found that inhibiting microglial activation or ablating microglia attenuated synapse loss in two different gp120 models [94]. Although the involvement of synapse pruning in the progression of synapse degeneration remains to be conclusively tested, these data, together with the emerging role of microglia in synapse pruning, suggest dysregulated microglial synapse pruning as a probable mechanism underlying HIV-induced synapse degeneration in the SDH pain neuronal circuit and the hippocampal and cortical neural circuits associated with HAND. From a conflicting perspective, it is also important to consider that impaired monocyte and macrophage phagocytosis has been reported in response to HIV [97]. A corresponding decrease in glial synaptic pruning could potentially lead to dysfunction in neuronal circuits through the loss of plasticity necessary for normal circuit function.

### 5.2. Potential Molecular Mechanisms of HIV-Induced Glia-Mediated Synaptic Pruning

Increased levels of complement proteins, including C1q and C3, have been reported in the CNS of patients with HIV [98]. The potential role of the complement system in HIV-induced synapse loss was investigated in a mouse model generated by intracranial injection of Tat protein. It was found that while global knockdown of C1q did not prevent synaptic degeneration, knockdown of C3 did result in increased synapse levels [93]. While C1q is necessary for initiation of the classical complement cascade, as mentioned above, C3 is also critical for developmental and disease-related synapse pruning and can trigger phagocytosis through the alternative complement pathway, independent of C1q [36,75,78]. Increased expression of C3 was found in the brain tissue of patients with HAND, and C3 expression by astrocytes was induced by HIV infection in vitro [99]. It is possible that C3 expressed by astrocytes, rather than C1q, mediates HIV-induced microglial synapse pruning. An additional synaptic pruning mechanism observed in the above diseases that could be relevant to HIV is the finding that IFN-γ produced by CD8+ T-cells contributes to the upregulation of synaptic pruning by microglia during WNV and ZIKV infection. This is because IFN- γ levels are increased in HIV patients’ brain tissue [100], and levels of CD8+ T cells expressing IFN- γ in the cerebrospinal fluid correlate with HAND in HIV patients [101]. It will also be important to consider how variations in TREM2 and APOE alleles between subjects might affect glial pruning behavior in diseases beyond AD, such as HIV. 

Little is known about the potential involvement of astrocytes in synapse pruning in the context of HIV-associated synapse loss. Astrocytes are reported to contribute to synaptic pruning during development and normal brain function, but dysregulated synaptic pruning by astrocytes leading to synaptic degeneration has not yet been seen. Yuan, et al., reported pain-associated synapse degeneration and astrocyte activation in the SDH of HIV patients [14]. These observations indicate an association between astrocyte activation and synapse degeneration in the SDH during the pathogenesis of HIV-related pain. Because gp120 is also implicated in this association [14], it will be interesting to directly test if reactive astrocytes in gp120 animal models manifest enhanced phagocytosis of synaptic components.

## 6. Conclusions

Glia-mediated synaptic pruning is vital for developing neuronal circuits and the plasticity of developed circuits. Disruption to this mechanism of structural plasticity may lead to circuit dysfunction (also referred to as maladaptive plasticity) and synapse degeneration [23]. Increased synaptic pruning has been revealed in several different diseases. Although it is possible that enhanced synaptic pruning during the development of CNS diseases could have a protective role for the CNS as a whole, such as preventing the spread of pathogens and/or toxic proteins, the resultant loss of synapses may disrupt neuronal networks and cause cognitive deficits. Multiple pathways, including the complement cascade, have been identified in glia-mediated synapse pruning in the contexts of development and diseases. It will be important to investigate the specificity and cooperation of the pathways in synapse pruning in different neuronal circuits. Synapse loss and glial activations are among the most prominent neuropathologies in the CNS of HIV patients and they are commonly considered crucial causes of neurological manifestations of HIV, such as HAND and pain. However, the pathogenic interaction between reactive glia and synapse loss has not been carefully studied. Recent work suggests a causal relation between HIV-associated glial reaction and synapse degeneration [93,94], although the underlying mechanism has not been completely elucidated. Glia-mediated synapse pruning provides a new and exciting perspective to investigate the pathogenic role of reactive glia in synapse degeneration in HIV-associated neurological complications in the CNS. The link between aberrant synaptic pruning and neurodegeneration has previously been investigated; however, establishing that aberrant synaptic pruning drives maladaptive plasticity and synapse loss in spinal cord pain circuitry, leading to HIV-associated pain, will be a novel discovery. Developing treatments that target the mechanisms of aberrant synaptic pruning will represent a major advance in not only the treatment of HAND and HIV-associated pain, but also other glia-mediated neurocognitive and pain disorders.

## Figures and Tables

**Table 1:** Glia-mediated synaptic pruning mechanisms in disease models.

Diseases	Microglia	Astrocytes
AD	C1q, C3, CR3 [65] ^1^	APOE [69] ^2^
	TREM2 [64] ^1^	
WNV	C3, C3aR [75] ^1^	No reported pruning activity
	IFN-γ [76] ^1^	
ZIKV	C3, C1q [78]	Phagocytosis of debris was reported, but no mechanism was reported [80]
	TNF-α [78]	
	IFN-γ [76] ^1^	
Amyotrophic lateral sclerosis (ALS)	C1q implicated [81]	No reported pruning activity
Parkinson’s Disease (PD)	Pruning was reported, but no mechanism was reported [82] ^1^	Neuronal Phagocytosis, no mechanism reported [82]
Multiple sclerosis (MS)	C3 [83] ^1^	No reported pruning activity

^1^ Phagocytosis confirmed via co-staining of glial marker with synapse marker and lysosomal marker (i.e., LAMP1), in vivo ^2^ or in vitro.
